# Understanding heart rate alarm adjustment in the intensive care units through an analytical approach

**DOI:** 10.1371/journal.pone.0187855

**Published:** 2017-11-27

**Authors:** Richard L. Fidler, Michele M. Pelter, Barbara J. Drew, Jorge Arroyo Palacios, Yong Bai, Daphne Stannard, J. Matt Aldrich, Xiao Hu

**Affiliations:** 1 University of California San Francisco, School of Nursing, San Francisco, CA, United States of America; 2 University of California San Francisco, Department of Anesthesiology and Perioperative Medicine, San Francisco, CA, United States of America; 3 San Francisco VA Medical Center, San Francisco, CA, United States of America; 4 University of California San Francisco, School of Medicine, San Francisco, CA, United States of America; 5 University of California San Francisco Medical Center, San Francisco, CA, United States of America; 6 University of California San Francisco, Department of Critical Care Medicine, San Francisco, CA, United States of America; Azienda Ospedaliero Universitaria Careggi, ITALY

## Abstract

**Background:**

Heart rate (HR) alarms are prevalent in ICU, and these parameters are configurable. Not much is known about nursing behavior associated with tailoring HR alarm parameters to individual patients to reduce clinical alarm fatigue.

**Objectives:**

To understand the relationship between heart rate (HR) alarms and adjustments to reduce unnecessary heart rate alarms.

**Methods:**

Retrospective, quantitative analysis of an adjudicated database using analytical approaches to understand behaviors surrounding parameter HR alarm adjustments. Patients were sampled from five adult ICUs (77 beds) over one month at a quaternary care university medical center. A total of 337 of 461 ICU patients had HR alarms with 53.7% male, mean age 60.3 years, and 39% non-Caucasian. Default HR alarm parameters were 50 and 130 beats per minute (bpm). The occurrence of each alarm, vital signs, and physiologic waveforms was stored in a relational database (SQL server).

**Results:**

There were 23,624 HR alarms for analysis, with 65.4% exceeding the upper heart rate limit. Only 51% of patients with HR alarms had parameters adjusted, with a median upper limit change of +5 bpm and -1 bpm lower limit. The median time to first HR parameter adjustment was 17.9 hours, without reduction in alarms occurrence (p = 0.57).

**Conclusions:**

HR alarms are prevalent in ICU, and half of HR alarm settings remain at default. There is a long delay between HR alarms and parameters changes, with insufficient changes to decrease HR alarms. Increasing frequency of HR alarms shortens the time to first adjustment. Best practice guidelines for HR alarm limits are needed to reduce alarm fatigue and improve monitoring precision.

## Introduction

Alarm fatigue is recognized as a serious health hazard for hospitalized patients [1–4]. Excess numbers of alarms, a very high false alarm rate, and a disproportionately small number of actionable alarms have desensitized clinical staff to alarms, even when they are critical in nature [5–9]. The physiological monitors are a major contributor to alarm fatigue in critical care areas such as the intensive care unit (ICU), post-anesthesia care unit (PACU), telemetry monitoring units, and emergency departments where patient conditions can deteriorate rapidly [10–12].

To combat the alarm fatigue crisis, many hospitals have taken the approach to change the level of alarm notification from “crisis” to “advisory”, or further alter them to inaudible “message” alerts [13–15]. Another approach is to widen the parameters on threshold-type alarms such as heart rate and respiratory rate, but this approach sacrifices sensitivity to changes in the patient status without follow up data on safety. Recognizing trends and combinations of alarms and laboratory values may increase precision in monitoring patient status [16–19]. More accurate HR detection methods could decrease false HR alarms due to artifacts, but would need to maintain or improve sensitivity, specificity, and meaningfulness, while minimizing the number of nuisance alarms and false alarms.

Data regarding alarm fatigue originates in descriptive analyses from quality improvement projects aiming to reduce numbers of alarms [5, 7–11,13–15]. There is a paucity of information regarding the psycho-behavioral aspects of adjusting physiological monitoring alarm settings. Since heart rate (HR) alarms are one of the most frequently occurring parameter alarms contributing to alarm fatigue [6, 12], this study aims to describe what relationships may exist between monitoring HR alarms and clinician behaviors in adjusting the HR alarm settings.

## Methods

### Design

This study is a secondary, quantitative, descriptive analysis of HR alarms using the UCSF Alarm Study data from five adult ICUs at a quaternary care hospital [6]. Examination of HR alarms from this dataset allowed description of responses to HR alarms. The UCSF Committee on Human Research approved the parent study [6] with waiver of patient consent since this data did not influence clinical care, allowing all consecutive patients inclusion in the study. Inclusion criterion was presence of HR alarms recorded in our database, without any excluded cases.

### Alarm data and vital signs files

Heart rate alarms in this study had institutional default settings of 50 beats/min (bpm) for lower and 130 (bpm) for upper limits. All HR alarms, vital signs, and physiologic waveforms were stored in a relational database (SQL server) using BedMasterEx software (Excel Medical, Jupiter, FL). Server query generated reports of the different types of HR alarms, including alarm messages, alarm start and end times, and date/time of patient transfer in and out to the ICU (see Table 1). This server maintains a continuous list of all alarms that occurred both prior to any customized settings, as well as alarms that occurred after adjustments. Custom software programming allowed parsing of alarm messages and extraction of additional parameters. Matlab® scripts were created to analyze these vital signs files to visualize trends of HR data.

**Table 1:** Example of a SQL server generated query for heart rate alarms used in these analyses.

MRN	AlarmMessage	AlarmStartTime	AlarmEndTime	UnitBed	TransferIn	TransferOut
000	HR 39 < 50	3/30/2013 17:21:50.50	3/30/2013 17:25:16.49	xxx	3/25/2013 14:19:00.00	3/30/2013 17:32:00.00
001	HR 181 > 130	3/8/2013 16:53:14.41	3/8/2013 16:53:18.55	yyy	3/8/2013 9:00:00.00	3/9/2013 18:29:00.00
002	HR 50 = 50	3/15/2013 14:06:49.00	3/15/2013 14:06:52.00	zzz	3/15/2013 13:35:00.00	3/16/2013 18:27:00.00

Heart rate values were calculated from raw ECG signals every two seconds by the GE Healthcare.

Heart rate values were calculated from raw ECG signals every two seconds by the GE Healthcare monitors (Solar 8000, software version 5.4). Heart rate threshold alarms are user configurable on the monitoring systems that was uniform across all ICU’s in this study. When a new patient is admitted to that bed, all alarm settings revert back to institutional default. Alarms for HR cannot be changed to a silent status or visual-only alert status by the clinical staff members, and settings for the alarm volume can only be done by institutional biomedical engineering and is uniform across all ICU’s in this study.

### Statistical analyses

Descriptive statistics depicted HR alarms and adjustments. Great variability existed in this diverse patient pool. As was demonstrated in the parent study, a minority of patients drive the majority of alarms for a particular unit. For this reason, we report the median (*Mdn*) as central tendency and the interquartile range (*IQR*) as variability. Descriptive analyses of alarms adjustments by day of the week and time of the day were carried out using Tableau® (version 9.1). Data were organized into two groups: one group of patients had adjustments to the HR alarm settings, and the other group HR alarms settings were unchanged. Mann-Whitney U tests compared time in the ICU, number of alarms/patient, and frequency of alarms/patient. Wilcoxon signed rank tests were used to analyze effects on frequency of the alarms before and after the first adjustment. Data were analyzed with 2-talied tests using SPSS (IBM, version 22).

## Results

### Descriptive analysis

The parent study (6) included 461 consecutive patients producing a total of 2,558,760 unique alarms in the 31-day study period: arrhythmia, 1,154,201; parameter, 612,927; technical, 791,632. There were a total of 381,560 audible alarms for an audible alarm burden of 187/bed/day. Of the 461 subjects, 337 (73.1%) patients that had any HR alarms produced 23,624 HR alarms for inclusion into these secondary analyses. Heart rate alarms produce the same audible warning sound (2 beeps) as many other conditions such as blood pressure parameter violations, greater than 10 premature ventricular complexes per minute, and respiratory rate being outside specified parameters. Data were collected from 5 different units including one cardiac, two mixed neurologic and neurosurgical, and two mixed medical/surgical intensive care units. Table 2 shows a synopsis of HR alarms broken down by unit demonstrating some unit-to-unit variation in the actual alarm settings and frequency, with the neurological and medical/surgical units sharing similar patients.

**Table 2:** Unit-based summary showing heart rate alarm metrics and parameter settings.

Heart Rate (HR) Alarms	10 ICC (Cardiac)16 beds	9 ICU (Med/Surg)16 beds	13 ICU (Med/Surg)16 beds	8 NICU (Neuro) 13 beds	11 NICU (Neuro) 16 beds	Total	Mean
**Total Alarms**	5898 (25.0%)	4084 (17.3%)	5438 (23.0%)	2163 (9.1%)	6041 (25.6%)	23624	4724.8
**Beds in unit**	16	16	16	13	16	77	
**# patients producing alarms over 30 days**	65	75	78	60	59	337 of 461 (73.1%)	
**Alarms/bed/day (mean)**	12.3	8.5	11.3	5.5	12.6		10.0
**Alarms/day/unit (mean)**	196.6	136.1	181.3	72.1	201.4		157.5
**Alarms/unit/hour (mean)**	8.2	5.7	7.6	3.0	8.4		6.6
**Alarm Duration (sec) [Range]**	22.1 [2–206]	22.0 {1–2240}	21.6 {1–3658}	21.3 {1–2968}	21.2 {1–2841}		21.6
**Lower Limit Alarms (% total per unit)**	2944 (49.9%)	1004 (24.6%)	1357 (25.0%)	931 (27.0%)	1934 (32.0%)	7823	1564.6
**Lower Limit Alarm (mode) [range]**	50 [40–80]	45 [40–70]	50 [36–75]	50 [39–60]	50 [5–155]		
**Upper Limit Alarms (% total)**	2954 (50.1%)	3080 (75.4%)	4081 (75.0%)	1579 (73.0%)	4107 (78.0%)	15454	3090.8
**Upper Limit Alarms (mode) [range]**	130 [90–150]	130 [95–190]	140 [110–180]	130 [75–170]	130 [5–190]		

There were 156 (46.3%) females and 181 (53.7%) males, mean age 60.3 years (SD 17.8). Patients spent a median of 2.0 (IQR = 5.0) days in the ICU. A total of 23,624 HR alarms occurred, 65.4% exceeding the upper limit and 34.6% below the lower limit. Of patients having HR alarms, the median number of HR alarms/patient was 18 (IQR = 72). It is unclear in this dataset whether patients with no HR alarms were at default settings or had been tailored. To discern high-frequency ECG signal interference as a source of HR alarm artifact, 26 alarms (0.11%) were for a HR above 251; 136 (0.58%) for a HR between 201 to 250. Of the remaining 14,290 high HR alarms, 12,240 (85.7%) were in the 101 to 150 range and 2,050 (14.3%) were in the 151 to 200 range.

From the patients that had any HR alarms, 51% had their HR parameters adjusted. There were 411 upper and 400 lower HR limit adjustments. Patients experienced a median of 8 (IQR = 24) HR alarms before having their limits adjusted, and a median of 17.9 (IQR = 45.1) hours passed until first HR alarm adjustment. When adjusted, the upper limit was increased by a median of 5.0 bpm (IQR = 10, range -55 to +60) and the lower limit was decreased by 1.0 bpm (IQR = 5, range -50 to +40).

### Heart rate alarm occurrence and adjustments

**Fig 1:**
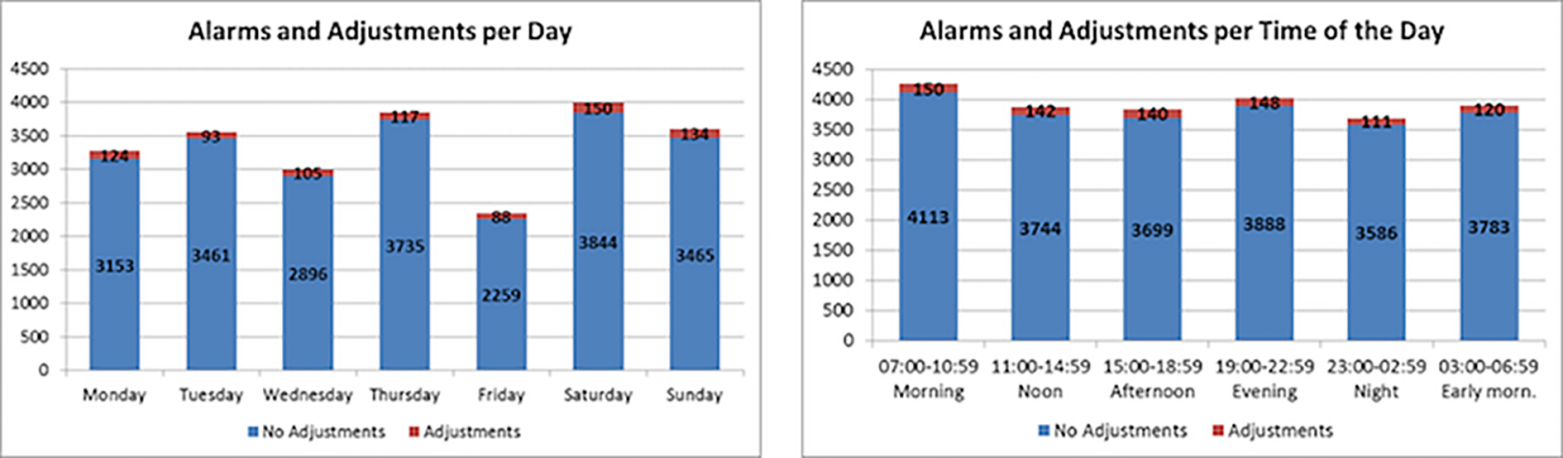
Alarm adjustment distribution: a) per day of the week, b) per time of the day.

Table 2 shows the number of heart rate alarms from each specific intensive care unit, and Fig 1A illustrates the distribution of HR alarm adjustments and HR alarms based on the day of the week. The occurrence of HR alarms is not equally distributed among the days of the week. Chi-square testing for goodness-of-fit analysis showed the observed frequency of alarms by day of the week differed significantly from a theoretically fair distribution of HR alarms by day of the week, [Chi-square (6, N = 23,624) = 562.7, p≤0.001], although the reason for this is uncertain, but may represent changes in patient census. Bivariate correlation analysis between the occurrence of HR alarms and adjustments to HR alarms settings per day of the week did not reach statistical significance (r = 0.71, p = 0.07).

Results on the analysis of the distribution of the occurrence of HR alarms and alarm adjustments during different times of day are illustrated in Fig 1B. Chi-square analysis showed observed distribution of HR alarms is different than the expected distribution of alarms during different times of day, [(5, N = 23,624) = 47.5, p≤0.001]. Bivariate correlation suggested a positive trend without statistical significance (r = 0.75, p = 0.08). Fig 1B suggests shift change may be associated with the largest numbers of adjustments in the HR settings at intervals starting at 7:00 am and 7:00 pm, as hospital policy states that settings should be reviewed at the time of nursing handoff.

### Analysis by alarms limits changed versus not changed

Table 3 summarizes descriptive statistics between groups with HR limits changed (n = 172) and those unchanged (n = 165). Mann-Whitney U tests showed significant differences in length of ICU stay (p<0.001, r = 0.45), alarms per patient (p<0.001, r = 0.61) and alarms/patient/day (p<0.001, r = 0.47).

**Table 3:** Descriptive statistics of patients with heart alarm parameters changed and unchanged.

	Stay in ICU (days)		# Alarms Per Patient		Alarms per patient per Day	
	Changed	Not Changed	Changed	Not Changed	Changed	Not Changed
median	3.73	1	64.00	4.00	15.21	2.98
IQR	7.77	1.35	117.75	15.00	25.49	8.13

**Fig 2:**
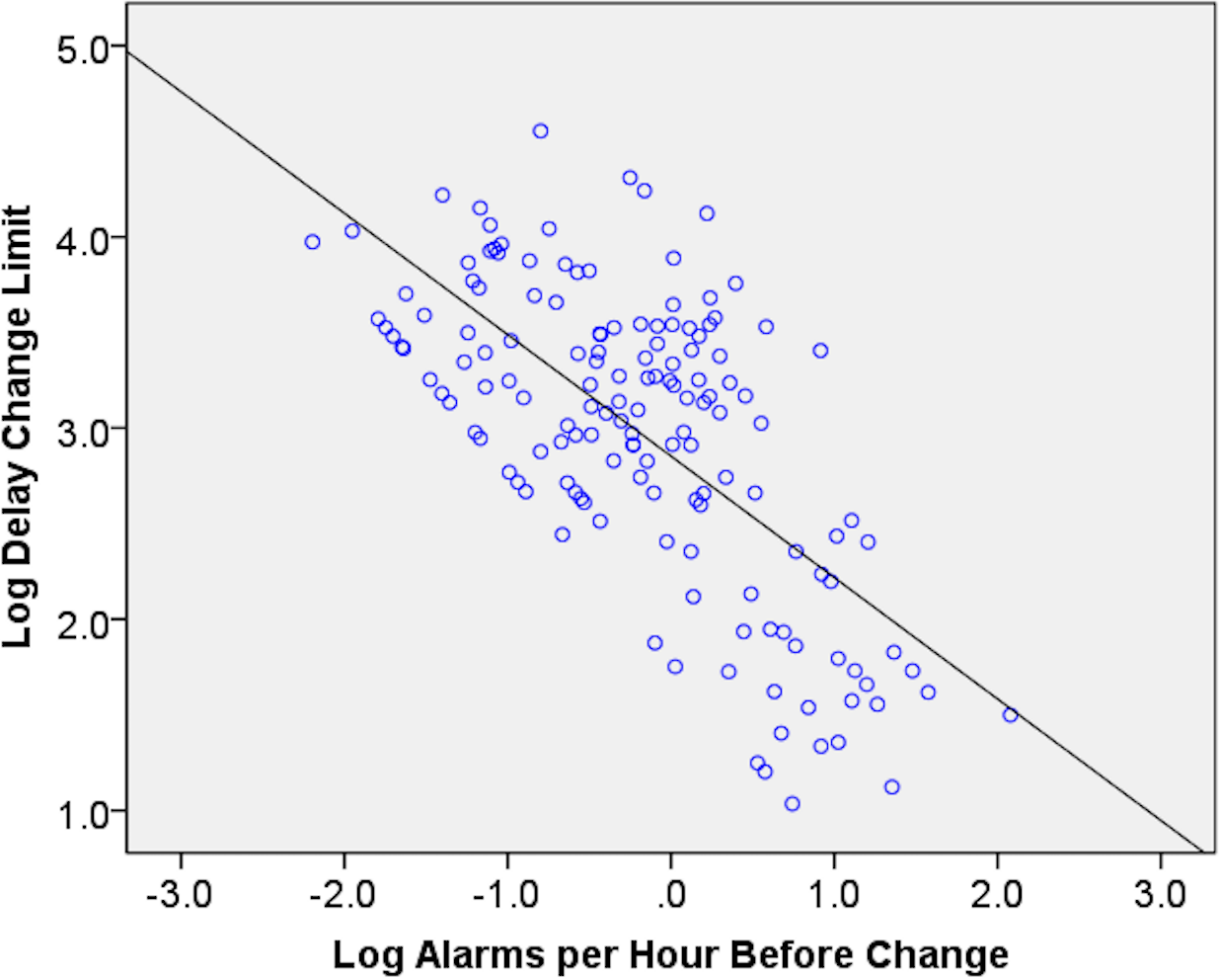
Relationship between number of alarms per hour before HR alarm setting change (log scale) and the hours from first alarm to change in HR alarm setting change (log scale). When the alarm frequency is higher, there was less delay in adjusting the HR alarm limits.

Patients without alarm limit adjustments had shorter ICU stays, fewer alarms, and alarms per day are lower. The difference on the rate of alarms/patient/day in Table 1 and Table 2 suggests that frequency of alarms may be a stimulus to change HR alarm settings. The binary logistic regression model was statistically significant ((1) = 32.6, p<0.001), explaining 12.3% (Nagelkerke R^2^) of the variance. Bivariate correlation analysis was performed on log-transformed data to investigate the frequency of alarms (alarms/hour before changing alarm limits) and delays on changing alarm limits. Fig 2 illustrates a relationship between the two variables, where higher numbers of alarms/hour was associated with a change in alarm limits.

Bivariate correlation corroborated significant negative correlation between the rate of alarms/hour (before the limit was changed) and the delay to adjust the alarm limits (r = -0.67, p<0.001).

The time prior to first HR alarm after admission was not a significant factor stimulating HR alarm adjustments. In patients with adjusted HR limits, the median time to the first alarm was 3.7 hours (IQR = 10.9), and was 4.6 hours (IQR = 10.4) for patients without changes to HR limits. These times were not different between the groups (r = 0.03, p = 0.56). This study is limited by not knowing whether patients that had HR alarm parameter adjustments on admission had no HR alarms for inclusion in our analyses.

### Frequency of alarms before and after first adjustment

**Fig 3:**
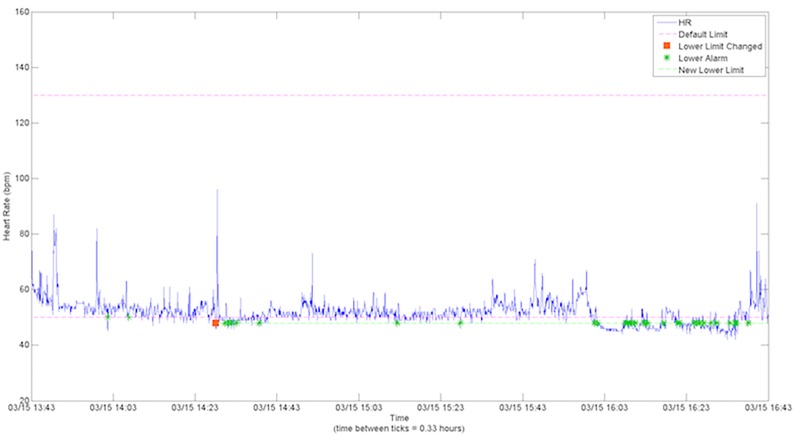
Occurrence of alarms and alarm adjustments in the HR vital files. The occurrence of alarms is indicated by a green * while the alarm adjustment is indicated by the orange square. The pink dotted lines represent the default lower and upper limits.

Analysis of HR data revealed that adjustments to HR limits did not reduce the number of HR alarms. Fig 3 shows two HR alarms occurred in a 5-minute period preceding adjusting the lower limit from 50 to 48 bpm. Nonetheless, this adjustment did not prevent 41 subsequent alarms over the next 2.6 hours, where multiple HR values were slightly less than 48 bpm. Wilcoxon signed-rank test showed that there was no significant difference on the frequency of alarms/hour before (Mdn = 0.46, IQR = 1.66) and after (Mdn = 0.65, IQR = 1.21) the alarm adjustment (p = 0.57).

## Discussion

Heart rate alarms are one of the most common alarms produced by the physiologic monitors. Tailoring HR alarms earlier and more meaningfully has potential to decrease HR alarms and improve sensitivity to patient condition changes. Although adjusting and personalizing alarm limits has been shown to reduce the occurrence of false alarms [14], this study shows that adjustments were not effective, exposing multiple levels of the problem. First, the fact that half of the patients’ alarm settings in this study were at hospital default the entire ICU stay is indicative that current practice is to maintain default HR alarm settings. It is important to note that many patients admitted to the hospital have a HR that is between the default thresholds; so some patients do not need to have these settings tailored. Jurisdictional issues may surround whether alarm settings are a nursing function, medical function, or collaborative [20]. Although institutional policy exists regarding tailoring alarm parameters, some variability in implementation obviously exists. Creating templates for ordering alarm settings on admission monitoring orders on the provider side may allow for initial diagnosis-based alarm settings. Empowering nurses with knowledge and protocols to make subsequent adjustments may be another potential solution. Additionally, improving education for staff has potential to optimize the monitoring experience for patients and clinicians. Optimization includes alarm reduction strategies that improve detection of patient changes, enhances accuracy with fewer false alarms, and demonstrates safety through outcomes focused research data.

Second, HR alarm adjustments were ineffective in reducing the numbers of HR alarms. Nurses changed HR alarm settings with wide variability and range, lacking consistency in the magnitude of adjustments. In most cases, the adjustment was too small to have a meaningful impact on the rate of HR alarms. There may be a need for several layers of parameter alarms. An example may be a lower HR alarm limit of below 50 bpm triggering a message (1 beep), but below 43 triggers a warning alarm (2 beeps), and below 35 is a crisis alarm (3 beeps continuously) until silenced by a staff member. A major technical shortcoming in monitoring equipment is the threshold-basis for HR alarms instead of a trend-based alarm. As an example, a HR change from 51 to 49 likely does not warrant an alarm, but when a patient experiences an abrupt HR change from 90 to 51, this likely signals a significant change in condition. Trending HR over time can provide some measure of the rapidity of the change in patient condition, and trend alarms have potential to improve monitoring precision.

Third, in this study, HR alarms were adjusted too late after admission to reduce the total number of HR alarms, especially in the first day of ICU admission. Although the AACN (American Association of Critical-Care Nurses) recommends customizing alarm settings within an hour of assuming care of a patient [21], the median time to make the first adjustment to HR alarm settings was 17.9 hours. Although this guidance has unclear scientific basis, it seems more reasonable to make adjustments to HR parameters within 1 hour, instead of 17.9 hours, to improve precision.

Fourth, alarm adjustments appear to be reactive rather than customized proactively. An increasing rate of alarms/day in patients was associated with an increasing likelihood of change in the alarm limits. It is a limitation in this study that patients that had no HR alarms may have benefitted from proactive parameter adjustments; however, our team did not have data to determine this. Moreover, our bivariate correlation analysis suggests that more alarms/hour stimulated alarm settings to be adjusted. Conservative adjustment of 1bpm (Mdn) was ineffective in low HR alarm reduction, although unknown factors contributed to such conservative adjustments. High HR alarm increases of 5 bpm (Mdn) were also ineffective at reducing high HR alarms. For altering the upper HR limit, there is likely more clinical latitude than lower HR limits to allow for a wider range of adjustment to still be in a "safe" heart rate range. For example, if the lower HR limit is set to 45 bpm, it may be crucial to know about a 5 bpm decrease; however a 20 bpm change may not as important when a high HR limit set to 150 instead of 130.

Fifth, HR alarms are definitely present over the course of the 24-hour day; however, distributions of alarms by day of week and time of the day are not uniform. Particular periods of time during the day had more high HR alarms likely due to clinician interventions, procedures, ambulation, and movement, and this represents one of several limitations of this study. Additional research that includes patient-clinician observation to validate the sources of alarms would be a worthwhile future study. The distribution of adjustments to HR alarms settings followed the occurrence of HR alarms. The largest number of adjustments to alarm settings appears to coincide with the time of nursing shift change at the study hospital (7 am and 7 pm), and further data granularity may help to alleviate this limitation in the future. The reason for the unequal distribution of HR alarm occurrence across days of the week is also unclear, but may represent changes in census due to the normal ebb and flow of patients into and out of the ICU.

The goals of HR alarms management should include strategies to not only reduce the total number of alarms and decrease false positive HR alarms, but also make HR alarms more meaningful.

Trending information regarding heart rate and diagnosis-specific data may improve clinicians' optimization of HR alarm limits, while improving the precision of HR monitoring [11, 22]. Some of the current alarms management strategies focus on the "symptom" of high numbers of alarms [9, 11, 13], but not the cause or contributing factors such as incorrect or inappropriate settings. Conceptually, adult HR alarm limits of 50 for the lower rate and 130 for an upper rate seem logical, representing a physiologically "normal" or acceptable range. An example of failure of the parameter approach to HR monitoring is atrial fibrillation with a rapid ventricular response, in a patient with a resting HR of 130–140 beats per minute upon admission. This patient would produce many alarms for exceeding the upper limit of 130 bpm. The default lower HR setting of 50 bpm would not detect either conversion to sinus rhythm or achievement of rate-control. In this case, setting the lower HR alarm threshold to a 100 bpm would signal treatment success. Also, setting the upper limit to 150 would reduce many alarms while detecting worsening condition or treatment failure. In this case, the precision approach to HR monitoring would identify the trend over time as significant [17, 22, 23].

Finally, it is important to note some of the limitations of this study. First, this is a retrospective study based only on the information provided when the alarms were triggered. It could be the case that there were some changes to the limits made by the clinicians but if they did not trigger any alarm we would not have any information about this. Second, the analyses in this study include data for only one month from a single academic quaternary care hospital. To improve generalizability, future analyses will benefit from data from longer periods of time to determine whether seasonal variation occurs in HR alarms, and further research needs to include data from wider variety of settings beyond this academic center. Third, the study presented a general overview of the incidence of HR alarms adjustments, but the results do not take into account the individual condition of patients. Moreover, due to the different possible health statuses from patient to patient, the data analyzed present great variability limiting generalizability. Longitudinal, multicenter studies are needed to make this type of data more meaningful.

## Conclusions

Our analyses suggest that adjustments to the alarm limits are not a current practice, with only half of the subjects in this study having HR alarm limits changed, and when done, the adjustments are typically reactive, ineffective, and too late. The problem has multiple components in need of improvement that could significantly reduce the occurrence of alarms while maintaining or improving monitoring precision. Educational strategies for clinical staff to optimize customizable settings based either on a patient disease state or current vital signs would be a significant step forward in improving ICU monitoring. Demonstration of safety by patient outcomes coupled with the reduction in nuisance alarms should be the expectation for future interventions aimed at alarm management and alarm fatigue reduction.

## Supporting information

S1 Dataset Deidentified March HR alarms.xlsx. This is a Microsoft Excel spreadsheet of the heart rate alarm metrics as described in the methods section.
(XLSX)
